# Sympatric Distribution of Giant Pandas and Red Pandas in Sichuan Province: Habitat Overlap Patterns and Implications for the Giant Panda's Umbrella Effect

**DOI:** 10.1002/ece3.73292

**Published:** 2026-03-17

**Authors:** Bin Feng, Weichao Zheng, Ying Fu, Mengyi Duan, Zhisong Yang, Hong Zhou

**Affiliations:** ^1^ College of Giant Panda China West Normal University Nanchong China; ^2^ Liziping Giant Panda's Ecology and Conservation Observation and Research Station of Sichuan Province Nanchong China; ^3^ Sichuan Academy of Giant Panda Chengdu China

**Keywords:** giant panda, habitat overlap, MaxEnt, red panda, umbrella species

## Abstract

In the context of limited biodiversity conservation resources, the umbrella species strategy is considered an efficient conservation approach. This study focuses on the sympatric distribution areas of the giant panda ( *Ailuropoda melanoleuca* ) and the red panda ( *Ailurus fulgens* ), examining whether giant panda–centered conservation frameworks may confer spatially coincident habitat protection for the red panda. Using the MaxEnt models, this study quantified the spatial overlap of suitable habitats and analyzed differences in habitat selection driven by environmental factors. All analyses were conducted within Sichuan Province, a key region of sympatry between the two species, and conclusions are restricted to this regional context. Results showed that the suitable habitat overlap between the two species was 9057 km^2^, accounting for 60.2% of the total red panda suitable habitat and 81.1% of the total giant panda suitable habitat, indicating a substantial degree of spatial overlap between the suitable habitats of the two species. Elevation was the primary factor driving habitat differentiation, with red pandas favoring elevations of 2000–3500 m and giant pandas preferring 1800–3200 m. Mixed forests formed the core habitat (53.45% of the overlap area). However, red pandas were more sensitive to farmland disturbance (21.5% contribution) and proximity to roads (5.8% contribution) than giant pandas, which also exhibited a significant response to agricultural encroachment (farmland contribution: 15.4%), and their high‐altitude habitats (> 3200 m) were weakly represented within the giant panda suitable habitat, revealing conservation gaps. These results suggest that giant panda–focused reserves spatially coincide with a considerable proportion of red panda suitable habitat within Sichuan Province, but attention should be paid to high‐altitude areas and disturbance‐sensitive zones, with priority given to managing mixed forests and shrublands to potentially improve the effectiveness of panda‐centered conservation strategies.

## Graphical abstract

This study quantified habitat overlap between giant and red pandas in their sympatric range using the MaxEnt model, confirming a significant umbrella effect for the red panda. However, conservation gaps exist, as red pandas are more sensitive to high‐altitude areas (> 3200 m) and anthropogenic disturbance, requiring refined management strategies for those specific areas.

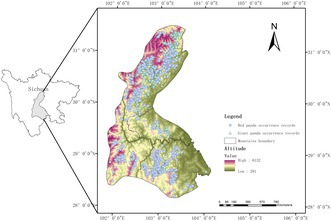

## 1 Introduction

Biodiversity conservation frequently faces the challenge of limited resources. To address this, the umbrella species strategy has emerged as an efficient conservation approach. Umbrella species are defined as those whose broad habitat requirements and ecological needs encompass those of coexisting species (Caro and Girling 2010; Caro and O'Doherty 1999). By prioritizing the protection of these key species and their habitats, conservation efforts can indirectly safeguard the biodiversity of sympatric species (Roberge and Angelstam 2004; Seddon and Leech 2008; Ward et al. 2020). The core assumption is that the intact ecosystems required by umbrella species (e.g., large contiguous forests, complex topography) will also meet the survival needs of other species (Fleishman et al. 2000).

The giant panda ( *Ailuropoda melanoleuca* ), a flagship species in global endangered species conservation, has often been regarded as a potential umbrella species because of its extensive habitat needs and reliance on intact forest ecosystems (Li et al. 2020). The giant panda has historically served as the primary focal species for large‐scale conservation planning in southwestern China, including the establishment of nature reserves and, more recently, the Giant Panda National Park (Wei et al. 2018). Evaluating whether this panda‐centered conservation framework confers collateral benefits to sympatric species, such as the red panda, therefore represents an applied conservation question, rather than a comparison of intrinsic umbrella potential between the two species (Li and Pimm 2016). Although earlier assessments indicated that giant panda reserves overlap spatially with a substantial proportion of suitable habitat for endemic birds and mammals (Xu et al. 2014), the universality of this umbrella function remains contentious. Notably, current panda‐centric reserve networks do not necessarily align with the conservation requirements of large carnivores like leopards ( *Panthera pardus* ) and wolves ( *Canis lupus* ), whose ranges have contracted despite long‐term protection policies (Li et al. 2020; Wang et al. 2021). Such discrepancies suggest that the umbrella effect is taxon‐specific rather than broad‐spectrum, limiting its utility for species with divergent ecological needs. Consequently, evaluating whether panda conservation frameworks benefit ecologically congruent species, such as the red panda, remains insufficiently evaluated.

The red panda ( *Ailurus fulgens* ), an endangered species sympatric with the giant panda, has often been assumed to benefit indirectly from panda conservation. Giant panda reserves may help restrict deforestation and road construction, maintaining the integrity of subalpine bamboo forests, which provide essential shelter and food resources for sympatric red pandas (Sharma and Belant 2010). Both species show substantial ecological niche overlap: they depend on subalpine mixed forests, use bamboo as a main food source, and share a narrow elevational range in the complex terrain of Southwest China (Fu 1999; Glatston 2011; Zhang et al. 2024). However, differences in responses to environmental factors may lead to divergence in habitat use (Pradhan et al. 2001; Thapa et al. 2018; Wei et al. 2000, 2004). For example, red pandas often occupy higher elevations (1500–4000 m), whereas giant pandas concentrate in mid‐elevation zones (1800–3400 m) (Liang et al. 2018). Such ecological heterogeneity may affect the actual protective effectiveness of the giant panda as an umbrella species for the red panda (Bai et al. 2023). Furthermore, their sensitivity to intensifying anthropogenic disturbances, such as farmland encroachment and transportation infrastructure, may differ substantially. This potential niche differentiation is a critical determinant in evaluating the implications and limitations of panda‐centered umbrella strategies (Xu et al. 2025). Despite growing interest in the umbrella role of the giant panda, existing studies have largely focused on spatial overlap or reserve coverage, often assuming that habitat congruence directly translates into effective protection for sympatric species. However, few studies have explicitly compared species‐specific responses to anthropogenic disturbance or jointly evaluated umbrella effectiveness from both spatial overlap and environmental driver perspectives. In this study, we address this gap by applying parallel MaxEnt models to quantify habitat overlap between giant pandas and red pandas in Sichuan Province, while explicitly contrasting their sensitivities to elevation and human disturbance factors, thereby identifying both the strengths and spatial limitations of the giant panda's umbrella effect.

Specifically, we targeted the Qionglai, Daxiangling, Xiaoxiangling, and Liangshan Mountains in Sichuan, the core area of sympatry. Our objectives were to: determine the extent to which giant panda habitats cover the red panda's core suitable habitat areas, identify key environmental factors (e.g., elevation, anthropogenic disturbance) driving differences in habitat selection, and determine the most frequently used habitat types by both species. Although the red panda population decline is well recognized, the core mechanisms driving habitat contraction and local extinctions remain unclear. Identifying the drivers of rapid habitat fragmentation and local extinction will provide scientific foundations for targeted conservation strategies, inject new perspectives into reserve management, and ultimately support the establishment of long‐term recovery plans and ecological security strategies.

## 2 Materials and Methods

### 2.1 Study Area

**FIGURE 1:**
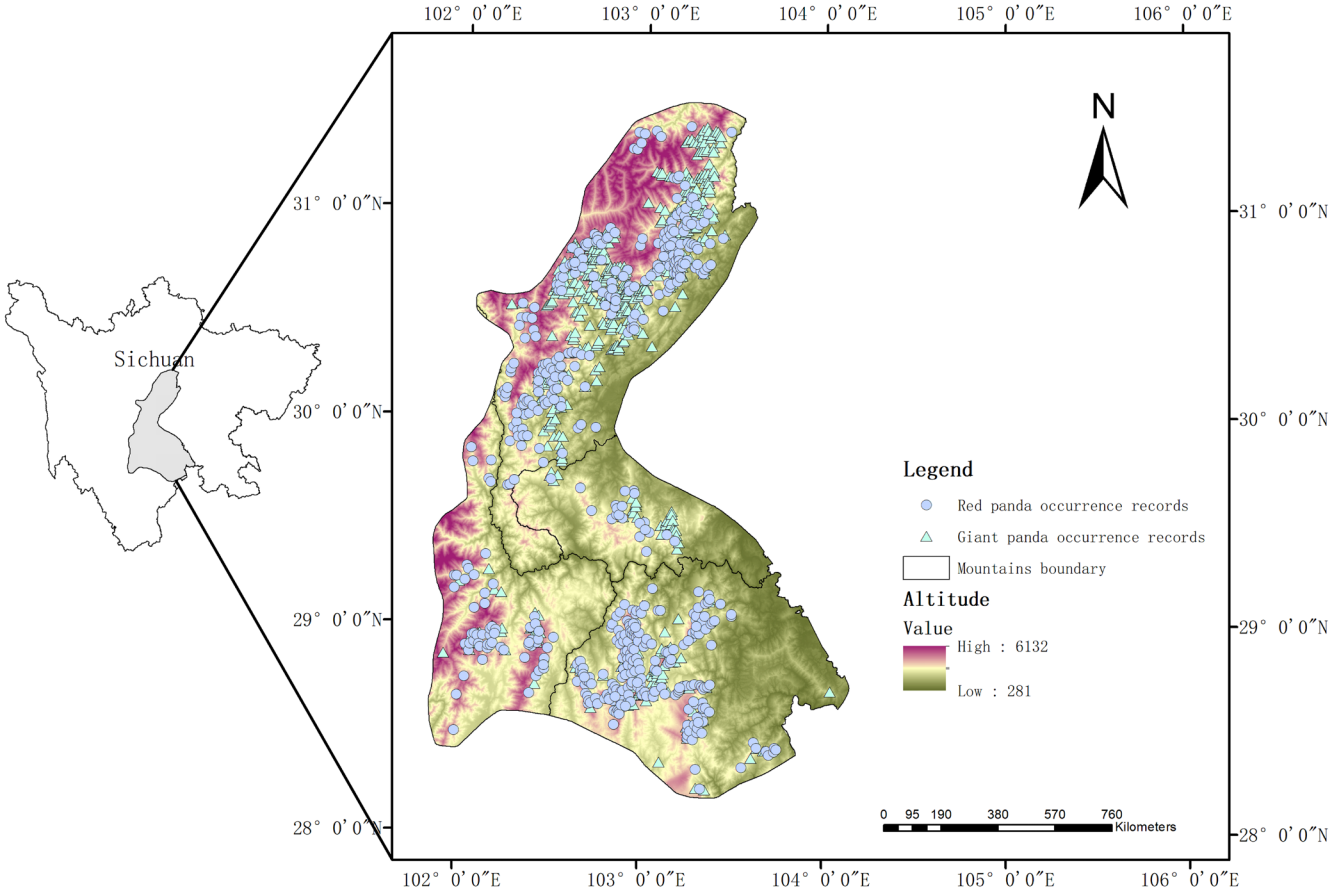
Study area and occurrence records.

This study focused on the overlapping distribution area of the giant panda ( *Ailuropoda melanoleuca* ) and the red panda ( *Ailurus fulgens* ) in southwestern China, covering the central and western regions of Sichuan Province (Figure 1). The complex topography, particularly the natural barriers formed by mountains and rivers, has created four relatively independent mountain ranges: Daxiangling, Xiaoxiangling, Qionglai, and Liangshan. Although geographically separated, these mountain ranges form an ecologically interconnected complex.

Notably, the Minshan mountain range, one of the primary distribution ranges of the giant panda and a key area in the Fourth National Survey on giant pandas and sympatric species, was excluded from this study because recent research indicates that red pandas have become extinct in this range (Ruan 2022; Ruan et al. 2024). The study area lies within the subtropical monsoon climate zone, but the terrain complexity results in pronounced vertical climatic differentiation. The extensive habitat requirements of the giant panda span diverse vegetation types and topographic conditions, and may overlap with environmental conditions relevant to other coexisting species. The distribution of red pandas in the area is similarly influenced by topography, climate, vegetation, and human disturbance.

### 2.2 Data Collection

To accurately assess giant panda distribution, we utilized data from the Fourth National Survey. This survey was conducted within the study area (Chinese State Forestry 2015). This survey recorded not only the distribution and abundance of giant pandas but also collected detailed data on sympatric species, including the red panda, providing an important data source for this study (Luo et al. 2020).

To reduce the potential effects of spatial autocorrelation, occurrence records were spatially thinned using the SDMtoolbox tool in ArcGIS, which employs a randomized algorithm to retain a single occurrence point within a 1 km radius (a distance comparable to the spatial resolution of our environmental predictors). After this spatial thinning process, 538 occurrence records for red pandas and 666 for giant pandas were retained for analysis. These data provided a robust basis for examining distribution patterns, habitat preferences, and interspecific habitat overlap.

### 2.3 Environmental Variables

Environmental variables were selected on the basis of the habitat preferences of both species and the spatial correlation among variables. A total of 19 bioclimatic variables were initially obtained from the WorldClim database. To reduce multicollinearity and avoid model overfitting, a two‐step variable screening procedure was applied. First, pairwise Pearson correlation coefficients were calculated among all bioclimatic variables across the study area. When two variables were highly correlated (|*r*| ≥ 0.8), only one variable was retained on the basis of its ecological relevance to panda habitat requirements and its contribution in preliminary model runs. Second, candidate variables were further evaluated using preliminary MaxEnt models and jackknife tests to assess their explanatory power. Through this screening process, three bioclimatic variables—annual mean temperature (BIO1), temperature seasonality (BIO4), and precipitation of the driest month (BIO14)—were retained, as they capture key aspects of thermal conditions and moisture stress relevant to bamboo‐dependent forest species while maintaining low collinearity with other predictors. Because spatially explicit bamboo distribution data are unavailable at a regional scale, forest type—particularly mixed forests—was used as a proxy for bamboo‐dominated habitats, an approach commonly applied in large‐scale habitat suitability modeling of bamboo‐dependent species (Hull et al. 2014). Eight variables were ultimately chosen: elevation, slope, distance to major roads, and proportions of farmland and forest, along with three climate variables (Table 1).

**TABLE 1:** Environmental variables used in habitat suitability modeling for the Chinese red panda.

Factor category	Variable	Description	Unit
Climate	BIO1	Annual mean temperature	°C
	BIO4	Temperature seasonality (SD × 100)	—
	BIO14	Precipitation of the driest month	mm
Terrain	ELEVATION	Mean elevation	m
	SLOPE	Mean slope	°
Land use	FARMLAND	Proportion of the cropland area	%
	FOREST	Proportion of the forest area	%
	ROAD	Distance to the main road	m

In this study, climate data were obtained from the WorldClim database (http://www.worldclim.org), which provides data at a spatial resolution of 30 arc‐seconds, approximately equivalent to a ground resolution of 1 km (Fick and Hijmans 2017). Land use data were sourced from the National Tibetan Plateau Data Center (https://data.tpdc.ac.cn), with a resolution ranging from 10 to 100 m. This dataset employs a hierarchical land use classification system, categorizing Sichuan into six first‐level classes: cropland, forestland, grassland, water bodies, urban, industrial, and residential areas, and unused land (Wang 2021).

Elevation data were derived from WorldClim, which originates from the Shuttle Radar Topography Mission (SRTM) elevation data (Fick and Hijmans 2017). On the basis of the Digital Elevation Model (DEM), slope data were extracted using ArcGIS (version 10.8). All environmental variables were processed in ArcGIS to generate raster data with a resolution of 1000 × 1000 m, which was subsequently exported in ASCII format for use in the MaxEnt models.

Land cover data were obtained from the MODIS/Terra + Aqua Land Cover Type Yearly L3 Global 500 m SIN Grid dataset, downloaded from the LP DAAC (https://lpdaac.usgs.gov/). The Terra and Aqua combined Moderate Resolution Imaging Spectroradiometer (MODIS) Land Cover Type (MCD12Q1) Version 6.1 data product provides global land cover types at yearly intervals. The MCD12Q1 Version 6.1 data product is derived using supervised classifications of MODIS Terra and Aqua reflectance data (Friedl and Sulla‐Menashe 2022). It should be noted that land‐cover variables represent landscape composition within grid cells rather than confirmed habitat use. In particular, shrubland and farmland were retained to characterize disturbance context and matrix composition, rather than being interpreted as preferred habitats. According to the International Geosphere‐Biosphere Programme (IGBP) classification scheme adopted by this product, ‘Woody Savannas’ specifically denotes lands with herbaceous and other understory systems, with forest canopy cover between 30%–60%, and where forest cover height exceeds 2 m. For analyses targeting the Sichuan region, this category has been redefined as ‘shrubland’.

### 2.4 Environmental Comparison Among Mountain Ranges

To characterize environmental heterogeneity among the four mountain ranges (Qionglai, Xiaoxiangling, Daxiangling, and Liangshan), we summarized key environmental variables within each mountain range polygon. Minimum, maximum, and mean elevation were derived from the digital elevation model (DEM). Farmland and forest proportions were calculated as the percentage of 1‐km land‐cover pixels classified as farmland or forest within each mountain range. Mean distance to roads was obtained by averaging road‐distance raster values across all pixels within each mountain range polygon. All environmental summaries were calculated at the same spatial resolution (1 km) used in the habitat suitability modeling to ensure consistency between descriptive statistics and model predictors.

### 2.5 Assessing Potential Umbrella Effects on the Basis of Habitat Overlap

The spatial overlap of suitable habitats between the two species was calculated, and the proportions of overlap within each species' total habitat were assessed. The spatial overlap percentage (SO) was computed as:

SO=A/B×100

where SO denotes the spatial overlap percentage, A represents the area of overlapping suitable habitats for both species, and B signifies the total suitable habitat area of the red panda. This methodology quantifies the extent to which suitable habitats identified for giant pandas spatially coincide with those of red pandas, thereby providing a quantitative description of spatial coincidence between suitable habitats under a panda‐centered conservation framework.

### 2.6 Modeling

MaxEnt version 3.4.4 was used to model the potentially suitable habitats for both species. To ensure model reproducibility and robustness, specific parameter settings were strictly defined. We generated 10,000 random background points (pseudo‐absences) from within the study area to characterize the available environmental space. The regularization multiplier was set to the default value of 1 to balance model goodness‐of‐fit with complexity and prevent overfitting. Given the substantial sample size for both species (> 80 records), we employed the ‘Auto’ feature class setting, which allowed the model to utilize all feature types (Linear, Quadratic, Product, Threshold, and Hinge) to rigorously capture complex non‐linear ecological responses. Model performance was primarily evaluated using the Area Under the Curve (AUC), whereas omission rates were monitored to verify that the model did not overfit the training data. Species occurrence points and environmental variables were used as inputs, with 75% of red panda data used for training and 25% for testing. Ten‐fold cross‐validation was applied, and the mean results across replicates were used to produce habitat suitability index (HSI) maps. It should be noted that MaxEnt outputs represent relative habitat suitability rather than confirmed species occupancy or population‐level protection.

HSI values range from 0 (least suitable) to 1 (most suitable). The model employs the Jackknife test to analyze the importance of ecological factors and utilizes the Area Under the Receiver Operating Characteristic Curve (AUC) to evaluate the accuracy of the MaxEnt models (Hanley and McNeil 1982). A higher AUC value indicates superior model performance. Model performance is considered poor when AUC < 0.7, moderate for 0.7 ≤ AUC ≤ 0.9, and excellent when AUC > 0.9 (Pearce and Ferrier 2000). The MaxEnt modeling results were imported into ArcGIS, where the maximum Youden index for each species was selected as the threshold to delineate suitable and unsuitable habitats for giant pandas and red pandas, respectively. The suitable habitats of both species were spatially overlaid to calculate their overlapping areas. This overlapping zone was further integrated with Land Cover data to quantify the proportional distribution of suitable habitats across different land cover types, thereby identifying the most frequently utilized habitat types for each species.

To ensure ecological realism and prevent physiologically unsuitable extreme high‐altitude terrain from artificially inflating the predictive importance of elevation, we applied a conservative upper elevation mask. Although the regional botanical treeline typically occurs at approximately 3600–3900 m, empirical occurrence records and previous studies indicate that both species may utilize sub‐alpine shrubland ecotones above the strict treeline for temporary foraging or dispersal (up to 4000 m; Liang et al. 2018). Consistent with our maximum recorded occurrence (4070 m for the giant panda), we therefore set the upper habitat boundary at 4200 m. This threshold retains all confirmed occurrence records and ecotonal habitats potentially accessible to both species, while excluding extreme alpine environments (e.g., permanent snow, glaciers, and alpine scree above 4200 m) that are physiologically unsuitable and outside the realized habitat niche. All background sampling and model training were constrained within this masked extent. We additionally verified that no occurrence records were removed by this elevation constraint.

## 3 Results

### 3.1 Habitat Suitability

**FIGURE 2:**
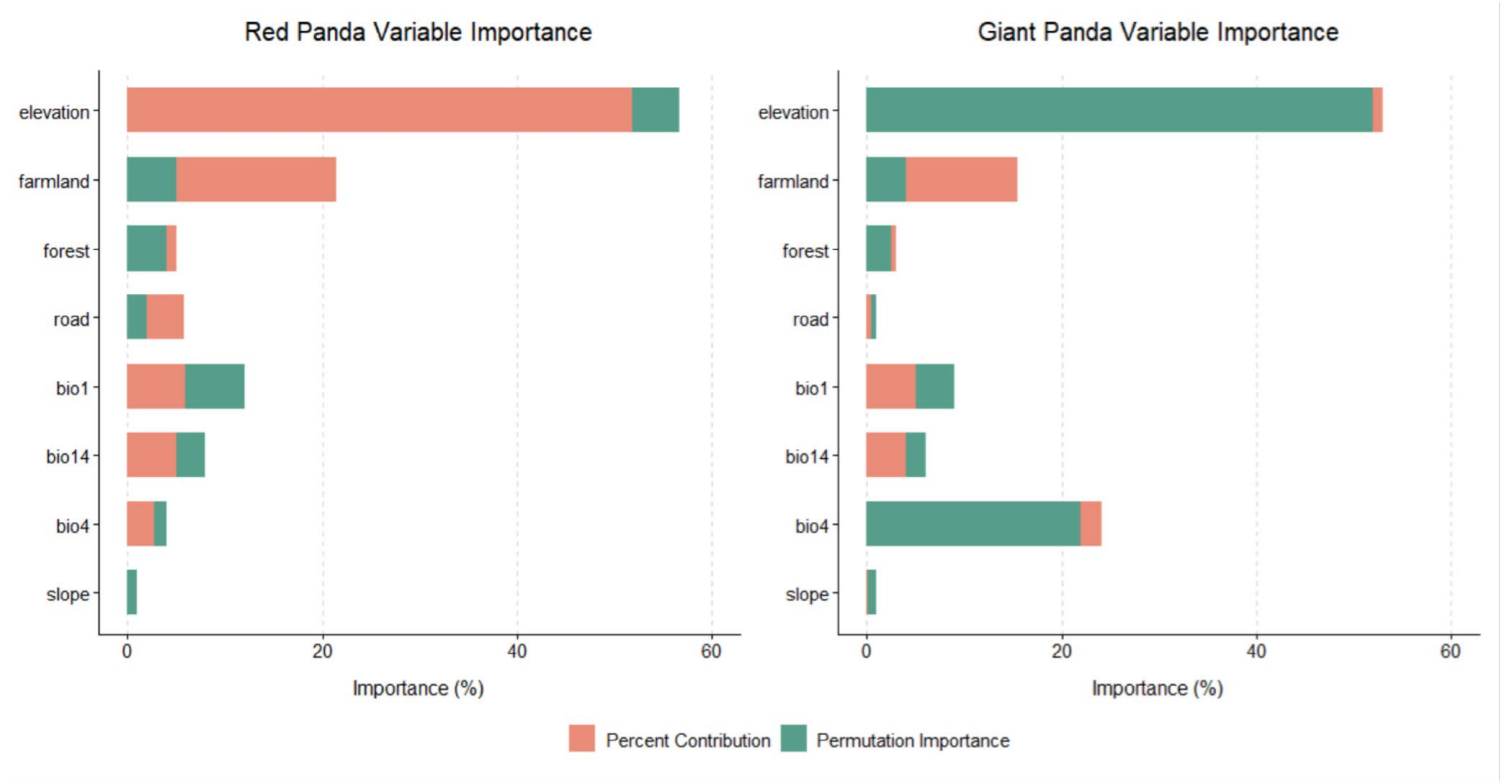
Permutation importance and contribution rates (%) of environmental variables to the MaxEnt models.

**FIGURE 3:**
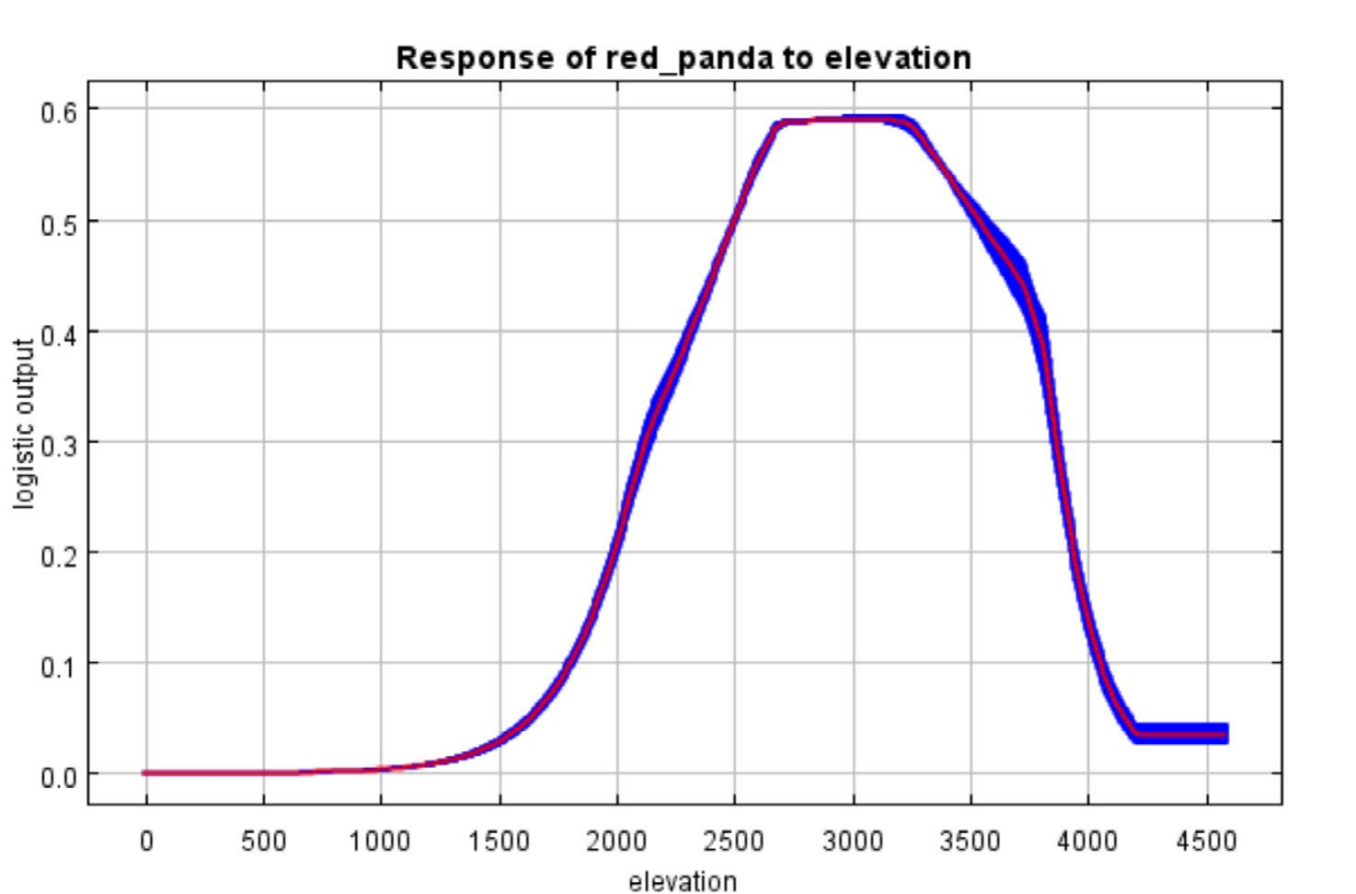
Response of red panda to elevation (m).

**FIGURE 4:**
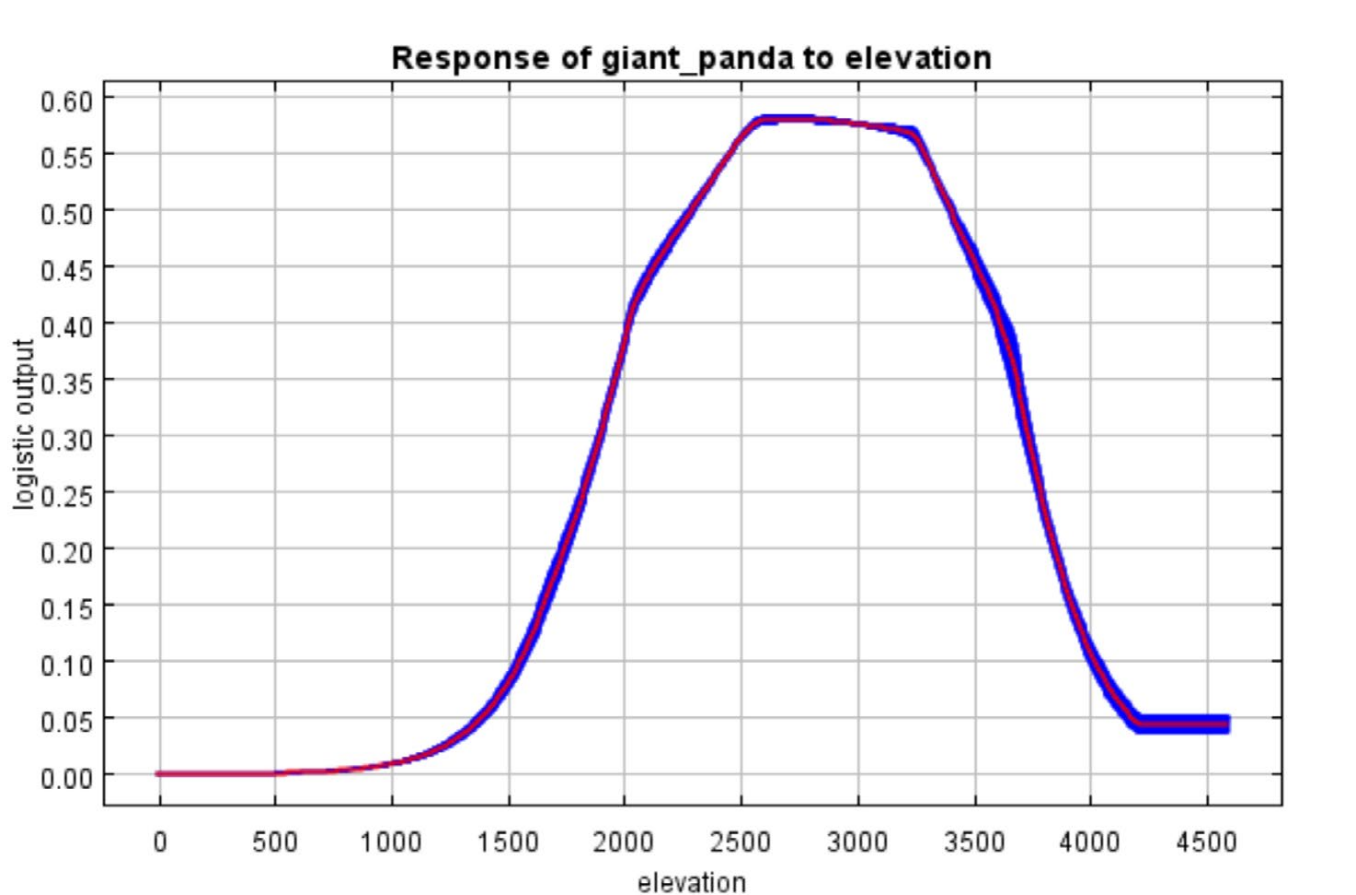
Response of the giant panda to elevation (m).

The average AUC value for the giant panda habitat suitability model was 0.846, and for the red panda model, it was 0.841. Both AUC values exceed 0.8, indicating good discriminatory ability of the models. MaxEnt models analysis on the basis of 10‐fold cross‐validation revealed that elevation dominated habitat suitability for both species. Elevation had a contribution rate of 51.8% to the red panda model and 44.9% to the giant panda model, with permutation importance values of 55.5% and 49.1%, respectively (Figure 2). Response curves indicated distinct elevational ranges associated with higher predicted suitability, where suitable habitat for red pandas was concentrated at 2000–3500 m (Figure 3), whereas giant pandas exhibited a downward shift to 1800–3200 m (Figure 4). Temperature seasonality (bio4) showed a contribution rate of 25.6% to giant panda habitat suitability—7.8 times higher than its contribution to the red panda model (3.3%). Human disturbance variables showed clear species‐specific differences. In the red panda model, farmland coverage exhibited a relatively high contribution rate (21.5%), indicating strong sensitivity to agricultural disturbance. In addition, distance to roads contributed 5.8% to red panda habitat suitability. In contrast, although giant pandas also exhibited a significant response to agricultural encroachment (farmland contribution: 15.4%), the red panda model showed even higher sensitivity to farmland coverage (21.5%). These results indicate that although both species are vulnerable to agricultural expansion, the red panda experiences a more pronounced impact from human‐mediated landscape changes.

**FIGURE 5:**
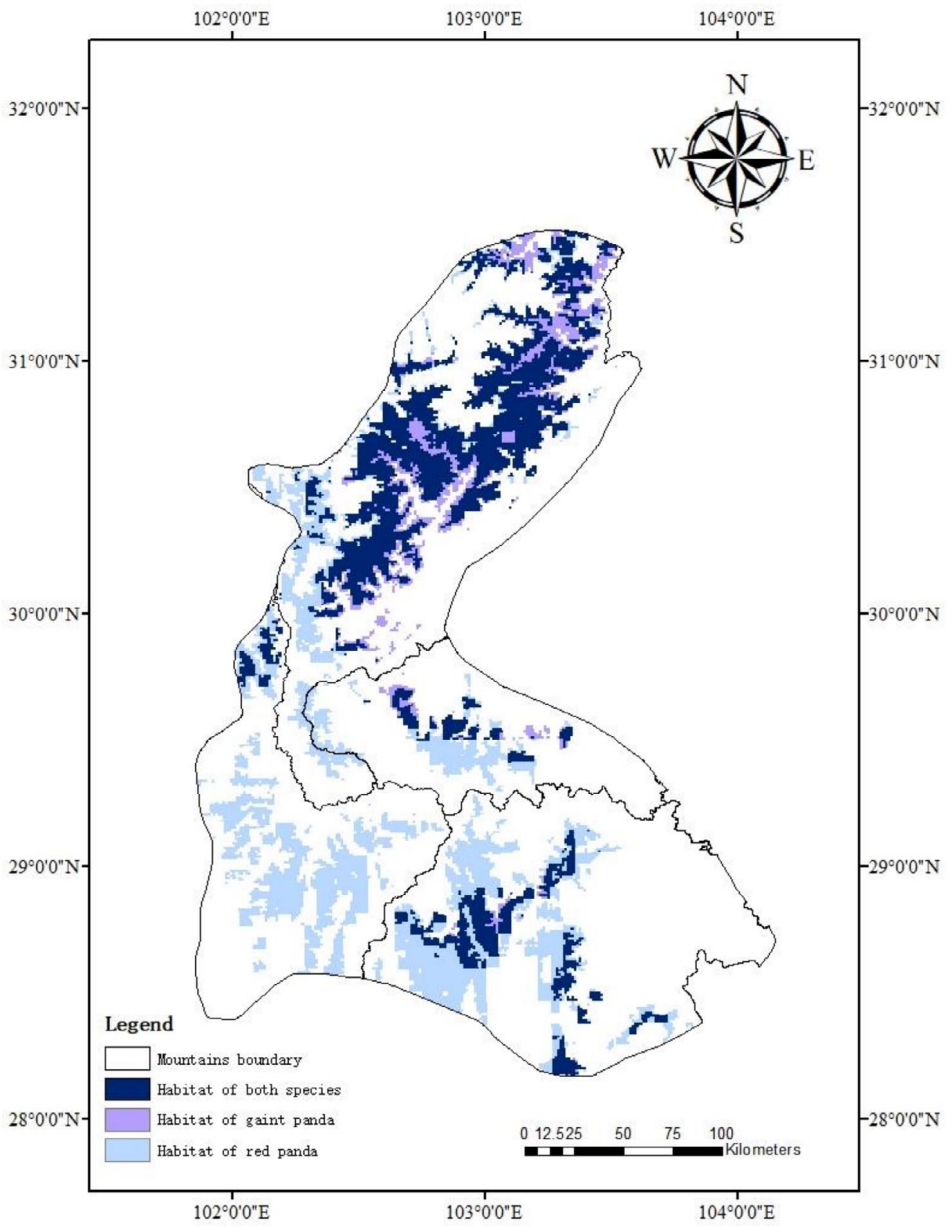
Habitat overlap between the red panda and giant panda.

On the basis of the HSI thresholds corresponding to the maximum Youden index (giant panda: 0.399300; red panda: 0.324900), continuous probability maps were classified into suitable/unsuitable habitats. GIS spatial analysis yielded the following measurements: total suitable habitat for red pandas covered 15,040 km^2^, whereas giant pandas occupied 11,161 km^2^, with an overlap area of 9057 km^2^ (Figure 5). The proportions of suitable habitat and overlapping areas between the two species are summarized in Table 2. The overlap constituted 60.2% of red panda habitat, indicating more than half of red panda habitats coexist with giant pandas. Conversely, it represented 81.1% of the giant panda habitat, suggesting a high degree of spatial containment of red panda habitat within the giant panda ranges. Overall spatial overlap accounted for 52.8% of the combined habitat area (17,144 km^2^), highlighting extensive sympatric distribution at the landscape scale.

**TABLE 2:** Habitat overlap statistics between the giant panda and red panda.

Indicator	Calculation formula	Value	Proportion (%)
Proportion of the red panda habitat	9057/15,040 × 100%	9057	60.2
Proportion of the giant panda habitat	9057/11,161 × 100%	9057	81.1
Proportion of the combined habitat	9057/17,144 × 100%	9057	52.8

### 3.2 Habitat Types

**FIGURE 6:**
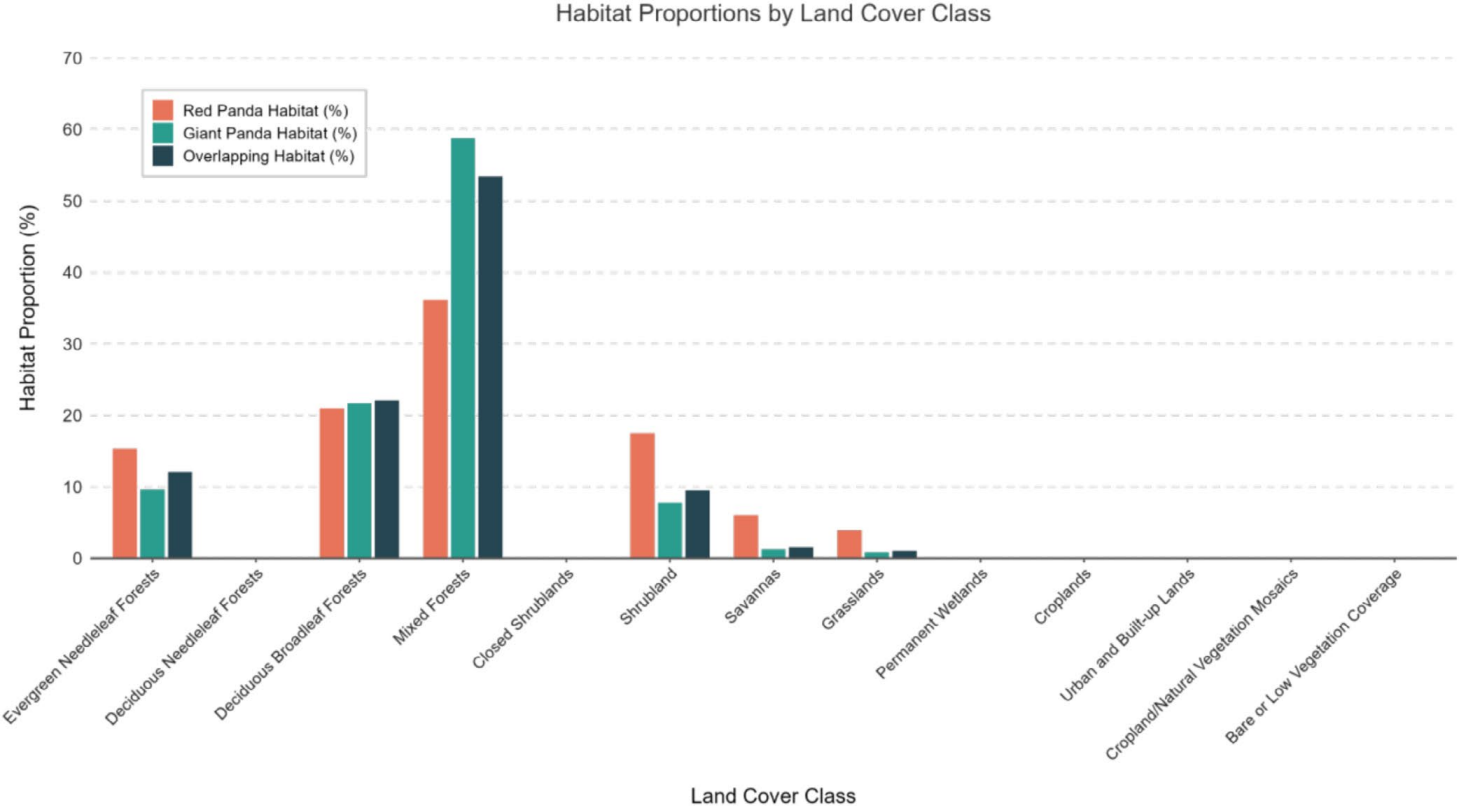
Proportional (%) distribution of land cover types.

Detailed analysis of habitat types revealed the composition characteristics of both species' habitats (Figure 6). Land cover classification showed that forest vegetation dominated habitats for both giant pandas and red pandas. Mixed forests formed the core habitat, accounting for 36.12% of red panda habitat, 58.71% of giant panda habitat, and 53.45% of the overlap area—making it the primary land cover type. Deciduous broadleaf forests were the second most dominant cover type, constituting 21.68% of giant panda habitat (slightly higher than 20.98% for red pandas) and 22.09% of the overlap area. Evergreen needleleaf forests covered 15.30% of red panda habitat, 9.57% of giant panda habitat, and 12.09% of the overlap area. Non‐forest vegetation types contributed relatively less to habitats. Shrublands followed, occupying 17.54% of red panda habitat, 7.75% of giant panda habitat, and 9.50% of the overlap area. Savannas and grasslands comprised 6.05% and 3.89% of red panda habitat, 1.30% and 0.83% of giant panda habitat, and 1.58% and 1.08% of the overlap area, respectively. Anthropogenically disturbed types (e.g., croplands, urban built‐up lands) each accounted for < 0.2% across all three habitat categories. Permanent wetlands and bare/low‐vegetation areas each represented < 0.05%. Vegetation composition in the overlap area aligned with overall habitats for both species, dominated by mixed and deciduous broadleaf forests, reflecting highly congruent preferences for forest ecosystems. However, certain land cover types (e.g., closed shrublands, cropland/natural vegetation mosaics) were absent in the overlap area.

### 3.3 Environmental Characteristics of the Four Mountain Ranges

To better understand the regional differences among mountain systems and evaluate the spatial heterogeneity of the umbrella effect, we summarized key topographic and anthropogenic characteristics for each range (Table 3). The Xiaoxiangling Mountains exhibit a high mean elevation (2790 m), slightly higher than Qionglai (2670 m). However, Xiaoxiangling experiences more intense road network encroachment (mean distance to roads: 5740 m). Conversely, the Daxiangling and Liangshan ranges show significantly lower mean elevations (1687 and 1845 m, respectively) but substantially higher farmland proportions (19.74% and 21.18%, respectively).

**TABLE 3:** Environmental characteristics of the four major mountain ranges in the study area.

Mountain range	Min elevation (m)	Max elevation (m)	Mean elevation (m)	Mean farmland proportion (%)	Forest proportion (%)	Mean distance to road (m)
Qionglai	585	6132	2670	6.99	56.48	8217
Xiaoxiangling	693	5972	2790	10.97	55.44	5740
Daxiangling	365	3968	1687	19.74	63.54	6433
Liangshan	281	4249	1845	21.18	57.30	7219

## 4 Discussion

### 4.1 Effectiveness of the Giant Panda as an Umbrella Species

Our quantitative analysis revealed a substantial degree of spatial coincidence between suitable habitats of the giant panda and the red panda within Sichuan Province. The results showed that 81.1% of the giant panda's suitable habitat overlapped with the suitable habitat for the red panda, indicating that a conservation network centered on the giant panda spatially coincides with a large proportion of red panda suitable habitat (Feng et al. 2023). These results provide quantitative evidence of habitat overlap under a panda‐centered conservation framework. The consistency of key driving factors further supports this conclusion. Both species are highly dependent on mixed forests at mid‐to‐high elevations (Zhang et al. 2006); 53.45% of their overlapping areas consist of mixed forests, and elevation is a shared dominant factor in the habitat suitability models of both species, with a contribution rate of 51.8% for the red panda and 44.9% for the giant panda (Figure 2). However, it is critical to interpret this dominance of elevation with caution. Elevation likely integrates multiple environmental and anthropogenic gradients in this region. In mountainous landscapes of southwestern China, lower elevations are typically associated with higher road density, farmland expansion, and historical human settlement, whereas higher elevations experience comparatively lower anthropogenic pressure. Therefore, the strong contribution of elevation in the models may partially reflect correlated human disturbance gradients rather than direct physiological constraints. As a result, elevation may mask the relative influence of individual disturbance variables, such as roads and farmland, whose independent contributions appear lower when elevation is included in the model. Because elevation acts as a composite proxy for multiple regional processes, the relative importance of predictors identified here should be interpreted within the specific socio‐ecological context of Sichuan Province. Caution is therefore needed when extrapolating these model relationships to other regions or to future scenarios where anthropogenic pressures may decouple from elevation gradients. Despite this caveat, this suggests partial similarity in environmental associations between the two species, particularly with respect to forest type and elevation, and mixed forests serve as their shared core habitat type. From the perspective of conservation strategies, the design of giant panda reserves (e.g., the delineation of core zones) spatially overlaps with approximately 60.2% of red panda suitable habitat, providing spatial evidence consistent with the umbrella species framework. The giant panda's requirement for large areas of contiguous forests (Zhang et al. 2011) and the red panda's preference for low‐disturbance environments form a synergistic effect; 81.1% of giant panda habitats overlap with red panda suitable areas, which is consistent with the conceptual expectation of spatial coverage underlying the umbrella species framework. Giant panda reserves encompass a substantial portion of environmentally suitable areas for red pandas, particularly in mixed forest zones at mid‐elevations (Ruan et al. 2021). It is critical to note that the total suitable habitat calculated for red pandas (15,040 km^2^) is substantially larger than that for giant pandas (11,161 km^2^). This fundamental difference in available habitat area explains the asymmetrical overlap percentages: although 81.1% of the giant panda's habitat is also suitable for the red panda, this shared area (9057 km^2^) conversely covers only 60.2% of the red panda's total suitable habitat. This suggests that the giant panda's realized niche may be largely nested within the broader fundamental niche of the red panda in this region. It also empirically highlights that a considerable portion (39.8%) of suitable red panda habitat exists outside the giant panda's range, directly quantifying the spatial limitations of the umbrella strategy. It should be noted that estimates of suitable habitat area and interspecific overlap are inherently sensitive to the choice of threshold used to convert continuous suitability outputs into binary maps. In this study, we applied the maximum Youden index, which balances sensitivity and specificity. According to our model results, the average logistic threshold on the basis of the Youden index (approx. 0.30 for red pandas) is higher than alternative thresholds such as the 10th percentile training presence (approx. 0.27). Consequently, using the 10th percentile threshold would imply a lower cutoff value, thereby classifying more marginal areas as suitable and potentially expanding the estimated suitable habitat area. Thus, our estimates on the basis of the Youden index likely represent a conservative estimate of spatial habitat overlap under the selected threshold. This threshold‐related uncertainty should be considered when interpreting the magnitude of the umbrella effect inferred from binary habitat maps.

### 4.2 Spatial Limitations of Panda‐Centered Habitat Overlap

Although the overall overlap rate is relatively high, there are still some conservation gaps, which are mainly reflected in the following aspects: Firstly, elevation gradient differences lead to coverage gaps. The suitable habitat elevation range for red pandas is 2000–3500 m, whereas that for giant pandas is 1800–3200 m, resulting in a 300‐m elevation difference between the two species. This means that red panda habitats at elevations above 3200 m are not covered by giant panda habitats. Such an elevation gradient difference may leave red pandas in high‐elevation areas without spatial coverage by the giant panda suitable habitat or reserves. Secondly, differences in sensitivity to anthropogenic disturbance exist. Red pandas are significantly more sensitive to anthropogenic disturbance than giant pandas (Chen et al. 2022). Specifically, in the red panda habitat suitability model, farmland coverage rate (contribution rate: 21.5%) and road distance (contribution rate: 5.8%) are the main anthropogenic disturbance factors; in contrast, the contribution rate of farmland in the giant panda habitat suitability model is 15.4% (with road proximity having minimal impact). This indicates that red pandas are more sensitive to disturbances from farmlands and roads, making their habitats more vulnerable to human activities—red pandas prefer habitats with less disturbance (Acharya et al. 2018; Bista et al. 2022; Zhang et al. 2011). This differential sensitivity to disturbance, compounded by the red panda's unique utilization of high‐altitude habitats (> 3200 m), creates a significant conservation gap. Consequently, red panda populations inhabiting high‐elevation zones that are also proximate to anthropogenic pressures may face localized threats that are not adequately mitigated by the current giant panda‐centric conservation network. Furthermore, against the backdrop of global climate change, red pandas may shift to higher elevations to adapt to environmental changes. However, existing reserves designed with giant pandas as the core may fail to provide sufficient shelter for red pandas, especially in areas above 3200 m. This suggests that when formulating conservation strategies, we need to consider the impact of climate change on species distribution, and appropriately adjust the scope and management measures of reserves to ensure that the future survival needs of red pandas are met.

These theoretical niche differences manifest distinctly across the region's complex geography. Our range‐specific topographic and environmental analysis (Table 3) reveals pronounced geographic heterogeneity among mountain systems, particularly in the southern and southwestern portions of the study area. The restricted distribution of giant pandas in the southwestern Xiaoxiangling Mountains is unlikely to be explained by elevation alone, as its mean elevation (2790 m) is comparable to that of Qionglai. Instead, Xiaoxiangling exhibits the shortest mean distance to roads (5740 m) among the four ranges, indicating relatively higher road density and potential habitat fragmentation. Such a landscape configuration may limit habitat continuity and reduce the availability of large, undisturbed forest patches required by giant pandas. Although elevation emerged as an important predictor in both models, the more restricted distribution of the giant panda within Sichuan appears to reflect a narrower combined tolerance to elevational and anthropogenic gradients. Compared to the red panda, giant pandas occupy a more constrained environmental window characterized by mid‐elevation forest habitats with lower levels of farmland encroachment and greater distance from roads. This narrower tolerance to disturbance‐related variables likely contributes to their more spatially limited distribution within the province. In contrast, the southern Liangshan Mountains display the lowest mean elevation (1845 m) and valley baseline (281 m), reflecting greater topographic accessibility. This accessibility has facilitated more intensive human land use, resulting in the highest farmland proportion (21.18%) among all ranges. Given the strong influence of farmland on red panda habitat suitability (21.5% contribution in the model), these southern systems are likely subject to elevated anthropogenic pressure affecting both species. Despite this, red pandas persist in these fragmented southern ranges likely due to their broader elevational tolerance and greater ecological flexibility. This wider environmental niche allows red pandas to occupy human‐modified landscapes provided that sufficient forest cover remains, whereas giant pandas—with their narrower disturbance tolerance—are spatially excluded. Collectively, these findings indicate that spatial differences in species distribution across mountain systems are shaped not solely by elevation, but by varying degrees of human disturbance and landscape fragmentation. In the southern and southwestern mountain ranges, conservation strategies should prioritize the restoration of low‐elevation forest patches, mitigation of agricultural expansion, and regulation of road development to maintain habitat connectivity. Such targeted management actions are particularly important in Xiaoxiangling and Liangshan, where suitable habitats are disproportionately exposed to anthropogenic pressure.

### 4.3 Priority in Habitat Type Management

Habitat type management occupies a core position in the conservation strategies for giant pandas and red pandas. Mixed forests account for 53.45% of the overlapping area, providing both species with critical food resources (bamboo) and shelter. Red pandas feed primarily on bamboo leaves (Reid et al. 1991), whereas giant pandas depend almost exclusively on bamboo as their primary food source (Wei, Feng, Wang, and Li 1999; Wei, Feng, Wang, Zhou, and Hu 1999). Given the key role of mixed forests in maintaining habitat integrity and ecological functions, they should be incorporated into the assessment indicator systems of nature reserves, and commercial logging and development activities must be strictly prohibited to ensure the sustained provision of their ecological functions (Linderman et al. 2005). Furthermore, the habitat composition analysis reveals that deciduous broadleaf forests (22.09% of overlap area) and evergreen needleleaf forests (12.09% of overlap area) also constitute significant components of the shared habitat. Therefore, although mixed forests represent the core priority, effective management strategies must extend to ensuring the integrity and connectivity of the entire forest matrix, which collectively accounts for 87.63% of the overlapping area. A holistic approach encompassing all these forest types is necessary to fully sustain the habitat requirements of both species. Shrublands make up 9.50% of the overlapping area. As critical ecological corridors connecting forests and meadows, their ecotone function is essential for maintaining ecosystem connectivity (Bennett 2003). To protect this area, fire prevention management should be strengthened—for instance, by establishing fire breaks—and grazing intensity must be strictly controlled to reduce damage to ecological corridors caused by human activities, thereby ensuring ecosystem connectivity (Bennett 2003). Low‐disturbance types (e.g., farmlands and built‐up areas) account for less than 0.2% of the study area, indicating that this ecosystem remains relatively intact. However, in habitat edge areas, vigilance is needed against the potential fragmentation effect of farmland expansion (currently accounting for 0.16%) on habitats (Urban et al. 1987), and measures should be taken to prevent its potential threats to the ecosystem. Therefore, in habitat management, priority should be given to the protection of mixed forests and shrublands, while guarding against the potential risks posed by low‐disturbance types. In conclusion, mixed forests, shrublands, and low‐disturbance areas are the priority areas for habitat management. By protecting the integrity of mixed forests, enhancing the ecological corridor function of shrublands, and preventing farmland expansion, the habitat quality for giant pandas and red pandas can be effectively maintained, providing a solid guarantee for their survival and reproduction.

## 5 Conclusion

This study quantified the habitat overlap pattern between giant pandas ( *Ailuropoda melanoleuca* ) and red pandas ( *Ailurus fulgens* ) in their sympatric distribution area in Sichuan using high‐precision habitat models, and systematically evaluated the potential umbrella effects of giant pandas for red pandas from a habitat‐based perspective. Key findings indicate that the giant panda conservation strategy exerts a significant yet incomplete umbrella effect on red pandas: the overlapping habitat area reaches 9057 km^2^, accounting for 60.2% of the total red panda habitat and 81.1% of the giant panda habitat. This confirms that the giant panda reserve network can spatially encompass more than half of the environmentally suitable habitat for red pandas—particularly in mixed forest‐dominated mid‐elevation regions (1800–3200 m), where their ecological needs are highly overlapping (with mixed forests comprising 53.45% of the overlapping area). However, the study also reveals key limitations: red pandas exhibit higher sensitivity to high elevations (> 3200 m) and anthropogenic disturbances (e.g., farmland expansion and road proximity), resulting in incomplete coverage of some red panda habitats. Although giant pandas, as an umbrella species, offer significant conservation coverage for red panda, the heterogeneous ecological needs (e.g., preferences for high elevations and greater disturbance sensitivity) of red pandas demand refined conservation strategies. Future efforts should focus on mechanistic analysis, gap management, cross‐species integration, and technological innovation to establish a resilient conservation network. This not only holds promise for reversing the declining trend of red panda populations but also contributes empirical evidence to global biodiversity conservation planning.

## Author Contributions

**Bin Feng:** conceptualization (equal), data curation (equal), formal analysis (equal), project administration (equal), software (equal), visualization (equal), writing – original draft (lead), writing – review and editing (equal). **Weichao Zheng:** conceptualization (equal), data curation (equal), investigation (equal), methodology (equal), supervision (equal), validation (equal), writing – review and editing (equal). **Ying Fu:** data curation (equal), investigation (equal), methodology (equal), software (equal), visualization (equal). **Mengyi Duan:** data curation (equal), formal analysis (equal), methodology (equal), validation (equal). **Zhisong Yang:** conceptualization (equal), data curation (equal), formal analysis (equal), investigation (equal), resources (equal), software (equal), supervision (equal), validation (equal). **Hong Zhou:** conceptualization (equal), data curation (equal), funding acquisition (equal), project administration (equal), resources (equal), software (equal), supervision (equal), validation (equal), writing – review and editing (equal).

## Funding

This work was supported by the Fundamental Research Funds of China West Normal University, 22kE028. National Natural Science Foundation of China, 32470538.

## Conflicts of Interest

The authors declare no conflicts of interest.

## Data Availability

The data that support the findings of this study are openly available in the Dryad repository at https://doi.org/10.5061/dryad.7h44j107p

## References

[ece373292-bib-0001] Acharya, K. P. , S. Shrestha , P. K. Paudel , et al. 2018. “Pervasive Human Disturbance on Habitats of Endangered Red Panda in the Central Himalaya.” Global Ecology and Conservation 15: e00420.

[ece373292-bib-0002] Bai, W. K. , J. D. Zhang , K. He , et al. 2023. “Implications of Habitat Overlap Between Giant Panda and Sambar for Sympatric Multi‐Species Conservation.” Wildlife Research 50: 820–826.

[ece373292-bib-0003] Bennett, A. 2003. Linkages in the Landscape; the Role of Corridors and Connectivity in Wildlife Conservation. IUCN.

[ece373292-bib-0004] Bista, D. , G. S. Baxter , N. J. Hudson , S. T. Lama , and P. J. Murray . 2022. “Effect of Disturbances and Habitat Fragmentation on an Arboreal Habitat Specialist Mammal Using GPS Telemetry: A Case of the Red Panda.” Landscape Ecology 37: 795–809. 34720409 10.1007/s10980-021-01357-w PMC8542365

[ece373292-bib-0005] Caro, T. , and S. Girling . 2010. Conservation by Proxy : Indicator, Umbrella, Keystone, Flagship, and Other Surrogate Species. Island Press.

[ece373292-bib-0006] Caro, T. M. , and G. O'Doherty . 1999. “On the Use of Surrogate Species in Conservation Biology.” Conservation Biology 13: 805–814.

[ece373292-bib-0007] Chen, X. , X. Wang , D. Kang , and J. Li . 2022. “Evaluating the Suitability and Overlap of Resting and Path Habitats of Giant Pandas in the Wanglang Nature Reserve.” Forests 13: 1795.

[ece373292-bib-0008] Chinese State Forestry . 2015. Results of the Fourth National Survey on the Giant Panda. Science Press.

[ece373292-bib-0009] Feng, B. , W. Bai , X. Fan , et al. 2023. “Species Coexistence and Niche Interaction Between Sympatric Giant Panda and Chinese Red Panda: A Spatiotemporal Approach.” Ecology and Evolution 13: e9937. 37091556 10.1002/ece3.9937 PMC10121233

[ece373292-bib-0010] Fick, S. E. , and R. J. Hijmans . 2017. “WorldClim 2: New 1‐Km Spatial Resolution Climate Surfaces for Global Land Areas.” International Journal of Climatology 37: 4302–4315.

[ece373292-bib-0011] Fleishman, E. , D. D. Murphy , and P. F. Brussard . 2000. “A New Method for Selection of Umbrella Species for Conservation Planning.” Ecological Applications 10: 569–579.

[ece373292-bib-0012] Friedl, M. , and D. Sulla‐Menashe . 2022. “MODIS/Terra+Aqua Land Cover Type Yearly L3 Global 500m SIN Grid V061.”

[ece373292-bib-0013] Fu, W. 1999. “Habitat Selection by Giant Pandas and Red Pandas in Xiangling Mountains.”

[ece373292-bib-0014] Glatston, A. R. 2011. Red Panda : Biology and Conservation of the First Panda. Academic Press.

[ece373292-bib-0015] Hanley, J. A. , and B. J. McNeil . 1982. “The Meaning and Use of the Area Under a Receiver Operating Characteristic (ROC) Curve.” Radiology 143: 29–36. 7063747 10.1148/radiology.143.1.7063747

[ece373292-bib-0016] Hull, V. , G. Roloff , J. Zhang , et al. 2014. “A Synthesis of Giant Panda Habitat Selection.” Ursus 25: 148–162.

[ece373292-bib-0017] Li, B. V. , and S. L. Pimm . 2016. “China's Endemic Vertebrates Sheltering Under the Protective Umbrella of the Giant Panda.” Conservation Biology 30: 329–339. 26332026 10.1111/cobi.12618

[ece373292-bib-0018] Li, S. , W. J. McShea , D. Wang , et al. 2020. “Retreat of Large Carnivores Across the Giant Panda Distribution Range.” Nature Ecology & Evolution 4: 1327–1331. 32747773 10.1038/s41559-020-1260-0

[ece373292-bib-0019] Liang, Y. , G. Wang , and H. Wang . 2018. “Analysis of Regional Environmental Characteristics of Giant Panda Activities Based on GIS: A Case Study of the Giant Panda National Park (Sichuan)[in Chinese].” Sichuan Forestry Science and Technology 39: 98–101.

[ece373292-bib-0020] Linderman, M. A. , L. An , S. Bearer , G. He , Z. Ouyang , and J. Liu . 2005. “Modeling the Spatio‐Temporal Dynamics and Interactions of Households, Landscapes, and Giant Panda Habitat.” Ecological Modelling 183: 47–65.

[ece373292-bib-0021] Luo, L. , H. Zhou , J. Tang , et al. 2020. “Ecological Differentiation of Giant Pandas and Their Sympatric Animals in Altitude Distribution.[in Chinese].” Journal of Mammalogy 40: 337–345.

[ece373292-bib-0022] Pearce, J. , and S. Ferrier . 2000. “Evaluating the Predictive Performance of Habitat Models Developed Using Logistic Regression.” Ecological Modelling 133: 225–245.

[ece373292-bib-0023] Pradhan, S. , G. K. Saha , and J. A. Khan . 2001. “Ecology of the Red Panda *Ailurus fulgens* in the Singhalila National Park, Darjeeling, India.” Biological Conservation 98: 11–18.

[ece373292-bib-0024] Reid, D. G. , H. Jinchu , and H. Yan . 1991. “Ecology of the Red Panda *Ailurus fulgens* in the Wolong Reserve, China.” Journal of Zoology 225: 347–364.

[ece373292-bib-0025] Roberge, J.‐M. , and P. Angelstam . 2004. “Usefulness of the Umbrella Species Concept as a Conservation Tool.” Conservation Biology 18: 76–85.

[ece373292-bib-0026] Ruan, T. 2022. “Study on the Changes in Distribution and Habitat Suitability of the Chinese Red Panda *(Ailurus styani)* in Sichuan.[in Chinese].”

[ece373292-bib-0027] Ruan, T. , H. Han , W. Wei , et al. 2021. “Habitat Suitability Evaluation for Giant Panda in Liziping National Nature Reserve, Sichuan Province.” Global Ecology and Conservation 30: e01780.

[ece373292-bib-0028] Ruan, T. , W. Wei , Z. Zhang , and H. Zhou . 2024. “Research on the Changes in Distribution and Habitat Suitability of the Chinese Red Panda Population.” Animals 14: 424. 38338067 10.3390/ani14030424 PMC10854785

[ece373292-bib-0029] Seddon, P. J. , and T. Leech . 2008. “Conservation Short Cut, or Long and Winding Road? A Critique of Umbrella Species Criteria.” Oryx 42: 240–245.

[ece373292-bib-0030] Sharma, H. P. , and J. L. Belant . 2010. “Threats and Conservation of Red Pandas in Dhorpatan Hunting Reserve, Nepal.” Human Dimensions of Wildlife 15: 299–300.

[ece373292-bib-0031] Thapa, A. , R. Wu , Y. Hu , et al. 2018. “Predicting the Potential Distribution of the Endangered Red Panda Across Its Entire Range Using MaxEnt Modeling.” Ecology and Evolution 8: 10542–10554. 30464826 10.1002/ece3.4526 PMC6238126

[ece373292-bib-0032] Urban, D. L. , R. V. O'Neill , and H. H. Shugart Jr. 1987. “Landscape Ecology.” Bioscience 37: 119–127.

[ece373292-bib-0033] Wang, F. , J. Winkler , A. Viña , et al. 2021. “The Hidden Risk of Using Umbrella Species as Conservation Surrogates: A Spatio‐Temporal Approach.” Biological Conservation 253: 108913.

[ece373292-bib-0034] Wang, J. 2021. “1:100,000 landuse dataset of Sichuan province (2000), National Tibetan Plateau Data, National Tibetan Plateau Data Center.”

[ece373292-bib-0035] Ward, M. , J. R. Rhodes , J. E. M. Watson , J. Lefevre , S. Atkinson , and H. P. Possingham . 2020. “Use of Surrogate Species to Cost‐Effectively Prioritize Conservation Actions.” Conservation Biology 34: 600–610. 31691376 10.1111/cobi.13430 PMC7318674

[ece373292-bib-0036] Wei, F. , R. Costanza , Q. Dai , et al. 2018. “The Value of Ecosystem Services From Giant Panda Reserves.” Current Biology 28: 2174–2180. 30008333 10.1016/j.cub.2018.05.046

[ece373292-bib-0037] Wei, F. , Z. Feng , Z. Wang , and J. Hu . 2000. “Habitat Use and Separation Between the Giant Panda and the Red Panda.” Journal of Mammalogy 81: 448–455.

[ece373292-bib-0038] Wei, F. , Z. Feng , Z. Wang , and M. Li . 1999. “Feeding Strategy and Resource Partitioning Between Giant and Red Pandas.” Mammalia 63: 417–430.

[ece373292-bib-0039] Wei, F. , Z. Feng , Z. Wang , A. Zhou , and J. Hu . 1999. “Use of the Nutrients in Bamboo by the Red Panda *(Ailurus fulgens)* .” Journal of Zoology 248: 535–541.

[ece373292-bib-0040] Wei, F. , M. Li , Z. Feng , Z. Wang , and J. Hu . 2004. “Sympatry of Giant and Red Pandas on YELE Natural Reserve, China.” In Giant Pandas, edited by L. Don and B. Karen , 189–200. University of California Press.

[ece373292-bib-0041] Xu, C. , X. Chen , Q. Dai , et al. 2025. “Multispecies View of the Effectiveness of the Giant Panda Conservation Programme.” npj Biodiversity 4: 34. 40897846 10.1038/s44185-025-00104-7 PMC12405537

[ece373292-bib-0042] Xu, W. , A. Viña , Z. Qi , et al. 2014. “Evaluating Conservation Effectiveness of Nature Reserves Established for Surrogate Species: Case of a Giant Panda Nature Reserve in Qinling Mountains, China.” Chinese Geographical Science 24: 60–70.

[ece373292-bib-0043] Zhang, Y. , W. Lei , W. Luo , Q. Dai , H. Han , and Y. Nie . 2024. “Comparison Study on the Trophic Niche of Red Pandas Using Stable Isotope Analysis.” Animals 14: 3512. 39682477 10.3390/ani14233512 PMC11639846

[ece373292-bib-0044] Zhang, Z. , R. R. Swaisgood , S. Zhang , et al. 2011. “Old‐Growth Forest Is What Giant Pandas Really Need.” Biology Letters 7: 403–406. 21227979 10.1098/rsbl.2010.1081 PMC3097871

[ece373292-bib-0045] Zhang, Z. , F. Wei , M. Li , and J. Hu . 2006. “Winter Microhabitat Separation Between Giant and Red Pandas in Bashania Faberi Bamboo Forest in Fengtongzhai Nature Reserve.” Journal of Wildlife Management 70: 231–235.

